# Gut dysbiosis associated with worse disease activity and physical function in axial spondyloarthritis

**DOI:** 10.1186/s13075-022-02733-w

**Published:** 2022-02-12

**Authors:** Jonas Sagard, Tor Olofsson, Elisabeth Mogard, Jan Marsal, Kristofer Andréasson, Mats Geijer, Lars Erik Kristensen, Elisabet Lindqvist, Johan K. Wallman

**Affiliations:** 1 Section of Rheumatology, Department of Clinical Sciences Lund, Lund University, Kioskgatan 5, 22185 Lund, Sweden; 2 Skåne University Hospital, Department of Rheumatology, Lund, Sweden; 3 Department of Immunology, EMV, Lund University, Lund, Sweden; 4 Department of Gastroenterology, Skåne University Hospital, Lund/Malmö, Sweden; 5 Sahlgrenska Academy, Institute of Clinical Sciences, Department of Radiology, University of Gothenburg, Gothenburg, Sweden; 6 Region Västra Götaland, Department of Radiology, Sahlgrenska University Hospital, Gothenburg, Sweden; 7 Section of Radiology, Department of Clinical Sciences Lund, Lund University, Lund, Sweden; 8 Parker Institute, Frederiksberg and Bispebjerg, Department of Rheumatology, Copenhagen University Hospital, Copenhagen, Denmark

**Keywords:** Spondyloarthritis, Ankylosing spondylitis (AS), Non-radiographic axial spondyloarthritis (nr-axSpA), Physical function, Microbiota, Disease activity, Dysbiosis, Gut inflammation, Inflammatory bowel disease

## Abstract

**Background:**

Based on clinical and genetic associations, axial spondyloarthritis (axSpA) and inflammatory bowel disease (IBD) are suspected to have a linked pathogenesis. Gut dysbiosis, intrinsic to IBD, has also been observed in axSpA. It is, however, not established to what degree gut dysbiosis is associated with axSpA disease severity.

The objective of this study was to compare gut dysbiosis frequency between controls, non-radiographic axial spondyloarthritis (nr-axSpA), and ankylosing spondylitis (AS) patients and investigate whether gut dysbiosis is cross-sectionally associated with axSpA disease activity, physical function, mobility, or pain.

**Methods:**

Gut dysbiosis was assessed by 16SrRNA analysis of feces from 44/88 nr-axSpA/AS patients (ASAS/mNY criteria) without inflammatory bowel disease (IBD) and 46 controls without IBD or rheumatic disease. The GA-map™ Dysbiosis Test was used, grading gut microbiota aberrations on a 1-5 scale, where ≥3 denotes dysbiosis. Proportions with dysbiosis were compared between the groups. Furthermore, standard axSpA measures of disease activity, function, mobility, and pain were compared between patients (nr-axSpA and AS combined) with and without dysbiosis, univariately, and adjusted for relevant confounders (ANCOVA).

**Results:**

Gut dysbiosis was more frequent in AS than controls (36% versus 17%, *p*=0.023), while nr-axSpA (25% dysbiosis) did not differ significantly from either AS or controls. Univariately, most axSpA measures were significantly worse in patients with dysbiosis versus those without: ASDAS-CRP between-group difference 0.6 (95% CI 0.2–0.9); BASDAI 1.6 (0.8–2.4); evaluator’s global disease activity assessment (Likert scale 0–4) 0.3 (0.1–0.5), BASFI 1.5 (0.6–2.4), and VAS pain (cm) 1.3 (0.4–2.2). Differences remained significant after adjustment for demographics, lifestyle factors, treatments, gut inflammation (fecal calprotectin ≥50 mg/kg), and gut symptoms, except for VAS pain. BASMI and CRP were not associated with dysbiosis.

**Conclusion:**

Gut dysbiosis, more frequent in AS patients than controls, is associated with worse axSpA disease activity and physical function, seemingly irrespective of both gut inflammation and treatments. This provides further evidence for an important link between disturbances in gastrointestinal homeostasis and axSpA.

**Supplementary Information:**

The online version contains supplementary material available at 10.1186/s13075-022-02733-w.

## Background

The link between spondyloarthritis (SpA) and inflammatory bowel disease (IBD) is well recognized. IBD patients are at increased risk of developing SpA, and the reverse is likewise true, with prevalence estimates of diagnosed IBD in axial SpA (axSpA) cohorts between 5 and 10% [1–3]. Several shared genetic risk loci are known [4, 5], and a common pathobiological pathway suggested [6, 7]. Additionally, approximately 50–60% of SpA patients display microscopic intestinal inflammation in biopsies of the ileum or colon, often reminiscent of Crohn’s disease [8–10].

IBD is associated with an altered intestinal bacteria composition [11], a condition known as intestinal or gut dysbiosis [12]. The intestinal inflammation characterizing IBD is thought to arise through a complex interplay between genetic, environmental, immunological, and gut microbial factors [13]. A variation of microbial aberrations has been reported in IBD, but a recurrent finding is a reduced species richness, particularly within the Firmicutes phylum [14–16]. Moreover, several studies have shown a decreased presence of *Fecalibacterium prausnitzii*, a species with protective, immune regulatory features, and increased abundance of *Escherichia coli*, possessing pathogenic properties [11, 17].

Recently, several studies have reported gut dysbiosis also in ankylosing spondylitis (AS) [18–23], whereas little is known about gut microbiota in non-radiographic axSpA (nr-axSpA) patients [24]. Furthermore, previous studies have linked intestinal inflammation, assessed either histologically [10, 25, 26], or estimated via elevated fecal calprotectin (F-calprotectin) levels [27, 28], to more active axSpA disease and worse prognosis. Yet, it remains largely unknown whether gut microbiota aberrations alone, independent of intestinal inflammation, is associated with more severe axSpA.

## Methods

The objectives of this study were to compare the frequency and degree of gut dysbiosis between well-characterized nr-axSpA and AS patients without known IBD and controls and to investigate whether gut dysbiosis is cross-sectionally associated with worse axSpA disease activity, physical function, mobility, or pain.

### Study population

Nr-axSpA and AS patients for the present study were enrolled from the population-based SPARTAKUS cohort of validated axSpA cases from southern Sweden. Comprehensive information about the SPARTAKUS cohort and its inclusion and diagnosis validation process has been published earlier [27]. Briefly, all patients from a defined geographical area of Skåne county, Sweden, with at least one outpatient visit to the Department of Rheumatology, Skåne University Hospital, with an ICD-10 diagnosis consistent with axial SpA (M45.9/M46.0/M46.1/M46.8/M46.9) during 2011–2014, were invited to enroll in the cohort. Among patients with a clinical diagnosis of undifferentiated SpA (M46.8/M46.9), only those reporting back pain ≥3 months with an onset before age 45 were eligible. Enrolled patients were extensively characterized through questionnaires, clinical examinations, blood/feces/urine sampling and imaging (X-ray and, if needed for the diagnosis validation/classification, also magnetic resonance imaging [MRI] of the sacroiliac [SI] joints), enabling classification into nr-axSpA or AS according to the Assessment of SpondyloArthritis international Society (ASAS) axSpA and modified New York criteria, respectively [29, 30]. For the present gut dysbiosis study, patients were consecutively enrolled from the SPARTAKUS cohort, whereby the first 150 subjects classified as nr-axSpA (*n*=50) and AS (*n*=100) without comorbid IBD, and who had provided a fecal sample (available for 88% of SPARTAKUS patients), were included. The study visits for these patients occurred 2015–2018.

A control group of 50 persons without rheumatic disease or IBD, and frequency-matched to the overall SPARTAKUS cohort by age and sex, was gathered among friends/colleagues/relatives of the authors. The controls followed a shortened protocol, including questionnaires and provision of blood and fecal samples (study visits during 2018).

Patients (*n*=18) and controls (*n*=4) reporting treatment with antibiotics within the last 3 months before they provided the fecal sample were excluded from the present study, with the final study population thus comprising 44 patients with nr-axSpA, 88 with AS and 46 controls.

### Gut microbiota analysis

Gut microbiota was analyzed using the GA-map™ Dysbiosis Test (Genetic Analysis, Oslo, Norway), a validated method designed to identify and grade gut dysbiosis in fecal samples by means of a pre-determined target approach, assessing the presence and abundance of a selected panel of 48 bacteria markers at different taxonomic levels and translating this into a dysbiosis index [31]. In brief, the test employs DNA probes, based on the 16SrRNA sequence in seven variable regions (V3–V9), to measure the abundance of bacteria according to the strength of fluorescent signal detection (probe signal intensity). The DNA probes included in the GA-map™ Dysbiosis Test were selected based on published observations regarding gut microbiota aberrations in IBD and IBS (irritable bowel syndrome), and in all target ≥300 bacteria in the six phyla Firmicutes, Proteobacteria, Bacteroidetes, Actinobacteria, Tenericutes, and Verrucomicrobia.

Based on bacterial composition and abundance by this sampling technique and applying a healthy Scandinavian control group as reference of normobiosis (normal microbiota composition), an algorithm was then developed to grade microbial aberrations from normobiosis, with a resulting Dysbiosis Index (DI) score of 1–5 [31]. A state of gut dysbiosis is considered present at DI ≥3 and severe dysbiosis at DI 5. A validation study of the GA-map™ Dysbiosis Test found dysbiosis (DI ≥3) in 16% of healthy volunteers and 74% of IBD patients [31]. Regarding the determination of gut dysbiosis, good agreement has also been demonstrated between the GA-map™ Dysbiosis Test and deep-sequencing by MiSeq Illumina sequencing technology [31].

The participants of the present study were instructed to collect their fecal sample as close in time to the study visit as possible and to store it in a −18°C freezer before delivery. After the visit, samples were stored frozen at −80°C until analysis (all samples were analyzed at the same time).

### Outcomes

Mean DI score 1–5, as well as the frequency of dysbiosis (DI ≥3), were compared between axSpA patients (AS and nr-axSpA combined) and controls. Similar comparisons were also performed separately for nr-axSpA versus controls, AS versus controls, and for nr-axSpA versus AS.

Distributions of the following standard axSpA measures were assessed in relation to DI score 1–5 and presence of gut dysbiosis (DI ≥3), respectively: measures of disease activity (Ankylosing Spondylitis Disease Activity Score using CRP [ASDAS-CRP]; Bath Ankylosing Spondylitis Disease Activity Index [BASDAI]; Evaluator’s Global disease activity assessment [EvalGlobal, Likert scale 0–4, signifying remission/low/medium/high/maximal]; CRP), physical function (Bath Ankylosing Spondylitis Functional Index [BASFI]), mobility (Bath Ankylosing Spondylitis Metrology Index [BASMI]), and pain (patient’s visual analog scale for pain [VAS pain]).

While, as outlined above, the focus of the present study was on the DI as a global measure of gut microbiota aberrations, in order to also allow for some comparisons with prior findings, exploratory analyses comparing the probe signal intensities for the 48 bacterial markers included in the GA-map™ Dysbiosis Test between the axSpA patients (nr-axSpA and AS combined) and controls are presented in Additional file 1.

### Statistics

Demographic, disease, and treatment characteristics were compared between groups by chi^2^-test, Fisher’s exact test or Mann-Whitney *U* test, as appropriate. Between-group comparisons of DI score 1–5 were performed univariately, as well as adjusted for age, sex, body mass index (BMI), and smoking (categorized as never smoker/quit ≥6 months ago/intermittent smoker or quit <6 months ago/everyday smoker) by analysis of covariance (ANCOVA). Due to the ordinal nature of the DI score 1-5, non-parametric bootstrapping with 10,000 iterations was used to calculate 95% confidence intervals (CI). BMI and smoking were adjusted for based on evidence that both are associated with intestinal microbiota alterations [32, 33]. The frequency of dysbiosis (DI ≥3) was compared between groups using chi^2^ analysis.

Comparisons of overall between-group differences in the various axSpA measures (regarding disease activity, function, mobility, and pain) between patients with different levels of DI score 1–5 were performed using one-way analysis of variance (ANOVA) or Kruskal-Wallis test, as appropriate. DI levels 4 and 5 were combined into one group, as there were too few cases with each score separately (*n*=3/*n*=5). Bootstrapped 95% CI were used for non-normally distributed measures (with normality assessed by Shapiro-Wilk test).

Comparisons of the axSpA measures (regarding disease activity, function, mobility, and pain) between patients with and without dysbiosis (DI ≥3) were performed univariately, as well as multivariately by ANCOVA, again applying bootstrapped 95% CI for non-normally distributed measures. The multivariate analyses were adjusted for age, sex, BMI, smoking (categorized as above), axSpA subtype (nr-axSpA/AS), HLA-B27 status, ongoing anti-TNF (tumor necrosis factor) therapy (yes/no), ASAS NSAID score for the preceding 3 months [34], gut inflammation as measured by F-calprotectin ≥50 mg/kg (yes/no; Calpro AS, Lysaker, Norway), and symptoms meeting the ROME III criteria for IBS (yes/no) [35]. Adjustment for axSpA subtype was considered relevant based on the numerically higher frequency of gut dysbiosis in AS, as compared to nr-axSpA, observed in the present study (see the “Results” section below) and the well-known differences in measures such as CRP and BASMI between the groups. Regarding treatments, anti-TNF agents are used to treat IBD and have been shown to affect gut microbiota in axSpA [20, 36], while NSAIDs can contribute to intestinal inflammation and also have potential to alter the gut microbiome [37]. Gut inflammation, as measured by F-calprotectin elevation, has been previously associated with both worse dysbiosis [19] and axSpA disease activity and function [27, 28] and was thus adjusted for to try to separate the effect of dysbiosis from that of inflammation. Finally, since a previously published study on the SPARTAKUS cohort found a high prevalence (30%) of IBS symptoms and that such symptoms were linked to comorbid fibromyalgia and worse patient-reported axSpA measures of disease activity, function, and pain [38], it was also deemed relevant to adjust for this, as gut dysbiosis is central to IBS [39].

To further ascertain that any observed associations between gut dysbiosis (DI ≥3) and axSpA measures (regarding disease activity, function, mobility, and pain) were linked to dysbiosis, and not to simultaneous intestinal inflammation or IBS, sensitivity analyses were performed excluding patients with elevated F-calprotectin (≥50 mg/kg) and IBS symptoms, respectively.

Welch’s *t* test with bootstrapped 95%CI (due to non-normal distributions; 10,000 iterations) was used for the exploratory comparisons of probe signal intensities for the 48 bacteria markers of the GA-map™ Dysbiosis Test between axSpA patients and controls.

Logarithmic values of ASAS NSAID score were used due to its skewed distribution. No imputations of missing data were performed. *p*<0.05 was considered statistically significant. SPSS, version 27 (IBM Corporation, NY, USA) was used for statistical analyses.

## Results

### Characteristics of the study population

Characteristics of the axSpA patients and controls are displayed in Table 1. Controls were well matched to the total patient group for age and gender. Compared to the AS group, nr-axSpA patients were on average younger and had shorter symptom duration, and a larger proportion were females. Ongoing or prior smoking was more common among AS patients than in the nr-axSpA or control groups. A numerically larger proportion of AS than nr-axSpA patients displayed gut inflammation (F-calprotectin ≥50 mg/kg), while presence of IBS symptoms was evenly distributed between the two axSpA groups. Similar proportions of nr-axSpA and AS patients received conventional synthetic and/or biologic DMARDs, with 41% anti-TNF use in both groups.

**Table 1:** Characteristics of the study population

	All axSpA (Nr-axSpA+AS)	Nr-axSpA	AS	Controls
	*n*=132	*n*=44	*n*=88	*n*=46
**Male sex,** ***n*** **(%)**	72 (55%)	17 (39%)	55 (63%) ‡	23 (50%)
**Age, years**	53 (13)	48 (12)	55 (13) ‡	51 (14)
**BMI, kg/m**^**2**^	27 (4.3)	27 (4.2)	27 (4.3)‡	25 (3.3) ‡‡
**Smoking status**				
Never smoker, *n* (%)	89 (67%)	35 (80%)	54 (61%)	32 (71%)
Quit smoking >6 months ago, *n* (%)	31 (24%)	7 (16%)	24 (27%)	11 (24%)
Intermittent smoker or quit <6 months ago, *n* (%)	5 (3.8%)	1 (2.3%)	4 (4.5%)	0
Every day smoker, *n* (%)	7 (5.3%)	1 (2.3%)	6 (6.8%)	2 (4.4%)
**Family history of SpA,** ***n*** **(%)**	58 (44%)	17 (39%)	41 (47%)	
**Symptom duration, years**	26 (14)	21 (11)	28 (14) ‡	
**HLA-B27 positive,** ***n*** **(%)**	114 (87%)	41 (93%)	73 (84%)	
**Back pain ≥3 months:**				
With onset <45 years, *n* (%)	127 (96%)	44 (100%)	83 (94%)	
Improved by exercise and not relieved by rest, *n* (%)	103 (79%)	33 (75%)	70 (81%)	
**Inflammatory back pain (ASAS definition),** ***n*** **(%)**	112 (85%)	37 (84%)	75 (85%)	
**Sagittal lumbar flexion (Modified Schober’s test), cm**	4.2 (1.6)	4.2 (1.2)	4.2 (1.8)	
**Lateral lumbar flexion, cm**^a^	14 (4.9)	15 (4.9)	13 (4.8) ‡	
**Chest expansion, cm**	4.8 (1.8)	5.0 (1.9)	4.7 (1.7)	
**Sacroiliitis on plain X-ray,** ***n*** **(%)**	88 (67%)	0 (0%)	88 (100%) ‡	
**SI joint MRI available,** ***n*** **(%)**	65 (49%)	25 (57%)	40 (45%)	
**SI joint bone marrow oedema on MRI,** ***n*** **(%)** ^b^	32 (49%)	9 (36%)	23 (58%)	
**Good response of back pain to NSAID,** ***n*** **(%)**	103 (78%)	33 (75%)	70 (80%)	
**Elevated CRP in the presence of back pain,** ***n*** **(%)**	82 (62%)	25 (57%)	57 (65%)	
**Peripheral arthritis,** ***n*** **(%)**	68 (52%)	26 (59%)	42 (48%)	
**Dactylitis,** ***n*** **(%)**	15 (11%)	9 (21%)	6 (6.8%) ‡	
**Heel enthesitis,** ***n*** **(%)**	57 (43%)	22 (50%)	35 (40%)	
**History of uveitis,** ***n*** **(%)**	46 (35%)	11 (25%)	35 (40%)	
**Skin and/or nail psoriasis,** ***n*** **(%)**	8 (6.1%)	3 (6.8%)	5 (5.7%)	
**IBD,** ***n*** **(%)**	0 (0%)	0 (0%)	0 (0%)	0 (0%)
**F-calprotectin, mg/kg**				
Mean (SD)	74 (131)	48 (55)	87 (155)	
Median (IQR)	33 (60)	29 (39)	39 (65)	
**Elevated F-calprotectin ≥50 mg/kg,** ***n*** **(%)**	46 (35%)	12 (28%)	34 (39%)	
**IBS symptoms,** ***n*** **(%)**^c^	43 (33%)	15 (34%)	28 (32%)	
**ASDAS-CRP**	1.8 (0.9)	1.9 (0.9)	1.8 (0.9)	
**BASDAI**	3.1 (2.2)	3.3 (1.9)	3.0 (2.4)	
**EvalGlobal, 0–4, median (IQR)**^d^	1 (0-1)	1 (0-1)	1 (0**–**1)	
**CRP, mg/l**	3.7 (5.3)	2.3 (2.4)	4.3 (6.1)	
**BASFI**	2.0 (2.1)	2.0 (1.7)	2.1 (2.2)	
**BASMI**	3.0 (1.4)	2.5 (1.1)	3.2 (1.5) ‡	
**VAS pain, cm**	3.3 (2.5)	3.4 (2.2)	3.2 (2.7)	
**ASAS 3-month NSAID score**	37 (44)	36 (44)	37 (44)	
**Use of proton-pump inhibitors last 3 months,** ***n*** **(%)**	53 (40%)	19 (43%)	34 (39%)	
**Ongoing csDMARD,** ***n*** **(%)**	24 (18%)	9 (20%)	15 (17%)	
Methotrexate, *n* (%)	14 (10.6%)	4 (9.1%)	10 (11.4%)	
Sulfasalazine, *n* (%)	8 (6.1%)	3 (6.8%)	5 (5.7%)	
Other csDMARD, *n* (%)	2 (1.5%)	2 (4.5%)	0 (0%)	
**Ongoing bDMARD,** ***n*** **(%)**	56 (42%)	19 (43%)	37 (42%)	
Adalimumab, *n* (%)	10 (7.6%)	2 (4.5%)	8 (9.1%)	
Certolizumab pegol, *n* (%)	11 (8.3%)	7 (15.9%)	4 (4.5%)‡	
Etanercept, *n* (%)	19 (14.4%)	5 (11.4%)	14 (15.9%)	
Golimumab, *n* (%)	5 (3.8%)	2 (4.5%)	3 (3.4%)	
Infliximab, *n* (%)	9 (6.8%)	2 (4.5%)	7 (8.0%)	
Secukinumab, *n* (%)	2 (1.5%)	1 (2.3%)	1 (1.1%)	

Mean (SD) if not otherwise stated. ^a^Mean of right and left lateral lumbar flexion. ^b^Previous or current SI joint bone marrow oedema according to the ASAS definition. ^c^Meeting the ROME III criteria for IBS. ^d^Likert scale 0–4, corresponding to Remission/Low/Medium/High/Maximal. ‡*p*<0.05 for between-group difference between nr-axSpA and AS by chi^2-^test/Fisher´s Exact test or Mann-Whitney *U* test, as appropriate. ‡‡*p*<0.05 for between-group difference between axSpA (nr-axSpA and AS combined) and controls by Mann-Whitney *U* test. Missing data, *n* (%): Symptom duration 1 (0.8%); HLA-B27 1 (0.8%); back pain improved by exercise but not by rest 1 (0.8%); chest expansion 1 (0.8%); F-calprotectin 2 (1.5%); IBS symptoms 1 (0.8%); ASDAS-CRP 16 (12%); BASDAI 7 (5.3%), EvalGlobal 5 (3.8%); CRP 15 (11%); BASFI 9 (6.8%); BASMI 2 (1.5%); VAS pain 2 (1.5%); Smoking, controls 1 (2.2%). *AS* ankylosing spondylitis, *ASDAS-CRP* ankylosing spondylitis disease activity score using CRP, *ASAS* Assessment of SpondyloArthritis international Society, *AxSpA* axial spondyloarthritis, *BASDAI* Bath ankylosing spondylitis disease activity index, *BASFI* Bath ankylosing spondylitis functional index, *BASMI* Bath ankylosing spondylitis metrology index, *bDMARD* biologic disease-modifying anti-rheumatic drug, *CRP* C-reactive protein, *csDMARD* conventional synthetic disease-modifying anti-rheumatic drug, *EvalGlobal* evaluator’s global assessment of disease activity, *HLA* human leukocyte antigen, *IBD* inflammatory bowel disease, *IBS* irritable bowel syndrome, *IQR* interquartile range, *MRI* magnetic resonance imaging, *nr-axSpA* non-radiographic axial spondyloarthritis, *NSAID* non-steroidal anti-inflammatory drug, *SD* standard deviation, *SI* sacroiliac, *SpA* spondyloarthritis, *VAS* visual analog scale

Regarding axSpA measures, BASMI values were significantly higher and CRP values numerically higher in the AS group, while other measures of disease activity, function, and pain were evenly distributed between the axSpA subtypes.

### Gut dysbiosis in axSpA and controls

**Fig. 1:**
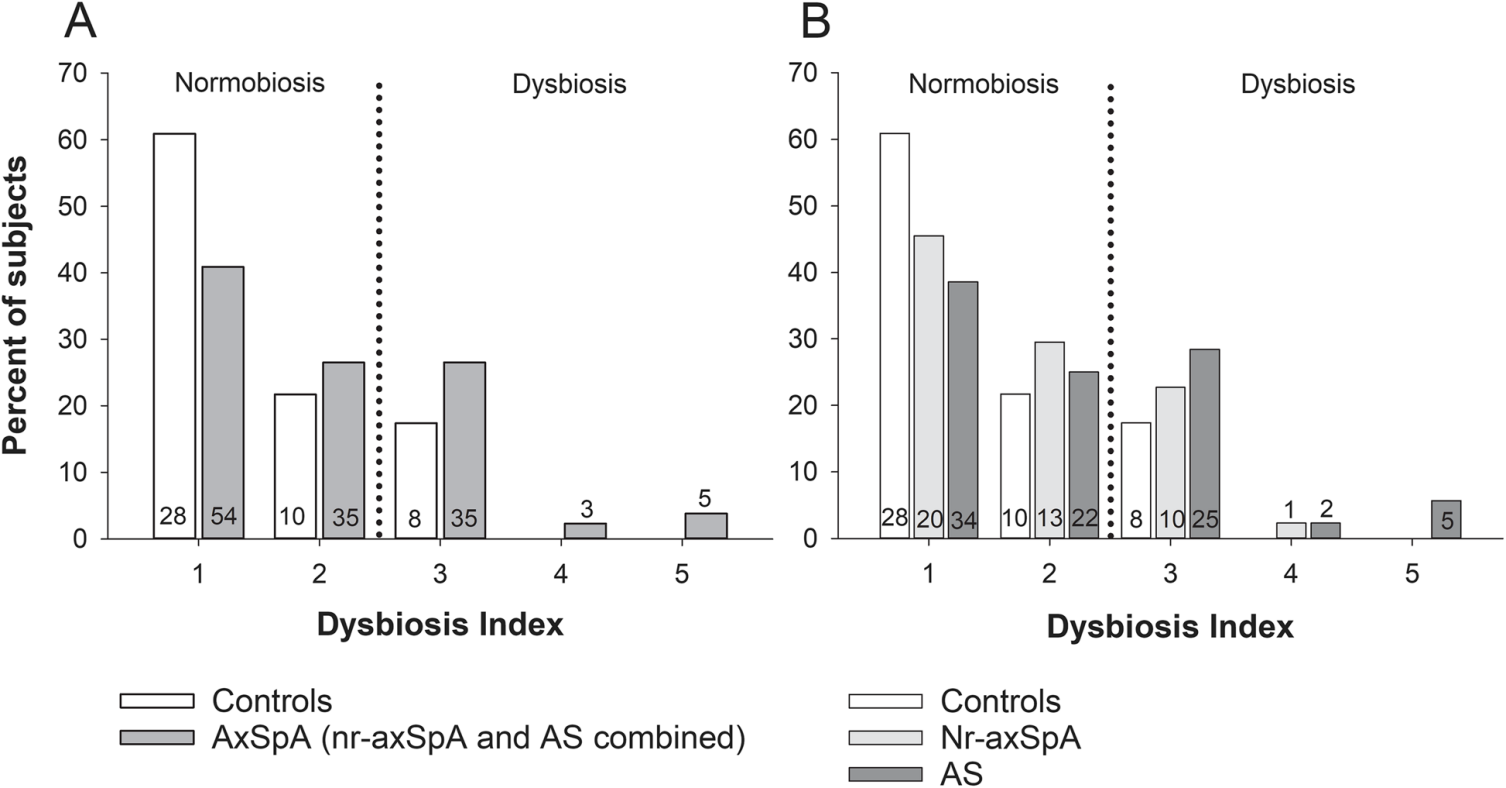
Dysbiosis index distributions among axSpA patients and controls. **A** Distributions of dysbiosis index (DI) score 1–5 among axSpA patients (nr-axSpA and AS combined) and controls. Unadjusted between-group difference in DI score 1–5: 0.45 (95% CI 0.16 to 0.74), *p*=0.003. Adjusted (for age, sex, BMI, and smoking) between-group difference in DI score 1–5: 0.34 (0.04 to 0.64), *p*=0.028. **B** Distributions of DI score 1–5 among nr-axSpA patients, AS patients, and controls. Nr-axSpA versus controls: unadjusted between-group difference in DI score 1–5: 0.25 (−0.09 to 0.60), *p*=0.154; adjusted (as above): 0.17 (−0.19 to 0.54), *p*=0.409. AS versus controls: unadjusted: 0.55 (0.22 to 0.88), *p*<0.001; adjusted: 0.43 (0.07 to 0.78), *p*=0.019. AS versus nr-axSpA: unadjusted: 0.30 (−0.06 to 0.64), *p*=0.098; adjusted: 0.20 (−0.18 to 0.56), *p*=0.286. Number of subjects per group presented at the bottom of (or above) the bars. AS ankylosing spondylitis, axSpA axial spondyloarthritis, BMI body mass index, CI confidence interval, DI dysbiosis index, nr-axSpA non-radiographic axial spondyloarthritis

DI score 1–5 was significantly higher among axSpA patients than controls (unadjusted between-group difference 0.45 [95% CI 0.16 to 0.74], *p*=0.003; adjusted between-group difference 0.34 [0.04 to 0.64], p=0.028); Fig. 1A). Yet, the frequency of gut dysbiosis (DI ≥3) was only numerically more common in axSpA versus controls (33% versus 17%; *p*=0.050). Of the 8 controls with dysbiosis, none displayed more pronounced aberrations from normobiosis, as measured by a DI of 4 or 5, whereas in the axSpA group this was observed in 19% of the dysbiotic patients (*n*=8 out of 43 patients with dysbiosis).

In our predefined subgroup analysis, both DI score 1–5 (results in Fig. 1B) and presence of gut dysbiosis were significantly higher in the AS group compared to controls (DI ≥3: 36% versus 17%; *p*=0.023). Conversely, for nr-axSpA, neither DI score 1–5 (results in Fig. 1B), nor the frequency of gut dysbiosis differed significantly from controls (DI ≥3: 25% versus 17%; *p*=0.377), although in both cases the nr-axSpA point-estimates fell between those of the control and AS groups. Analogously, no significant between-group differences in DI score 1–5 (results in Fig. 1B) or presence of dysbiosis (*p*=0.189) were detected between the nr-axSpA and AS groups.

Results of the exploratory analyses comparing probe signal intensity for the 48 bacterial markers of the GA-map™ Dysbiosis Test between axSpA patients and controls are presented in Additional file 1.

### AxSpA measures in relation to dysbiosis index levels

**Fig. 2:**
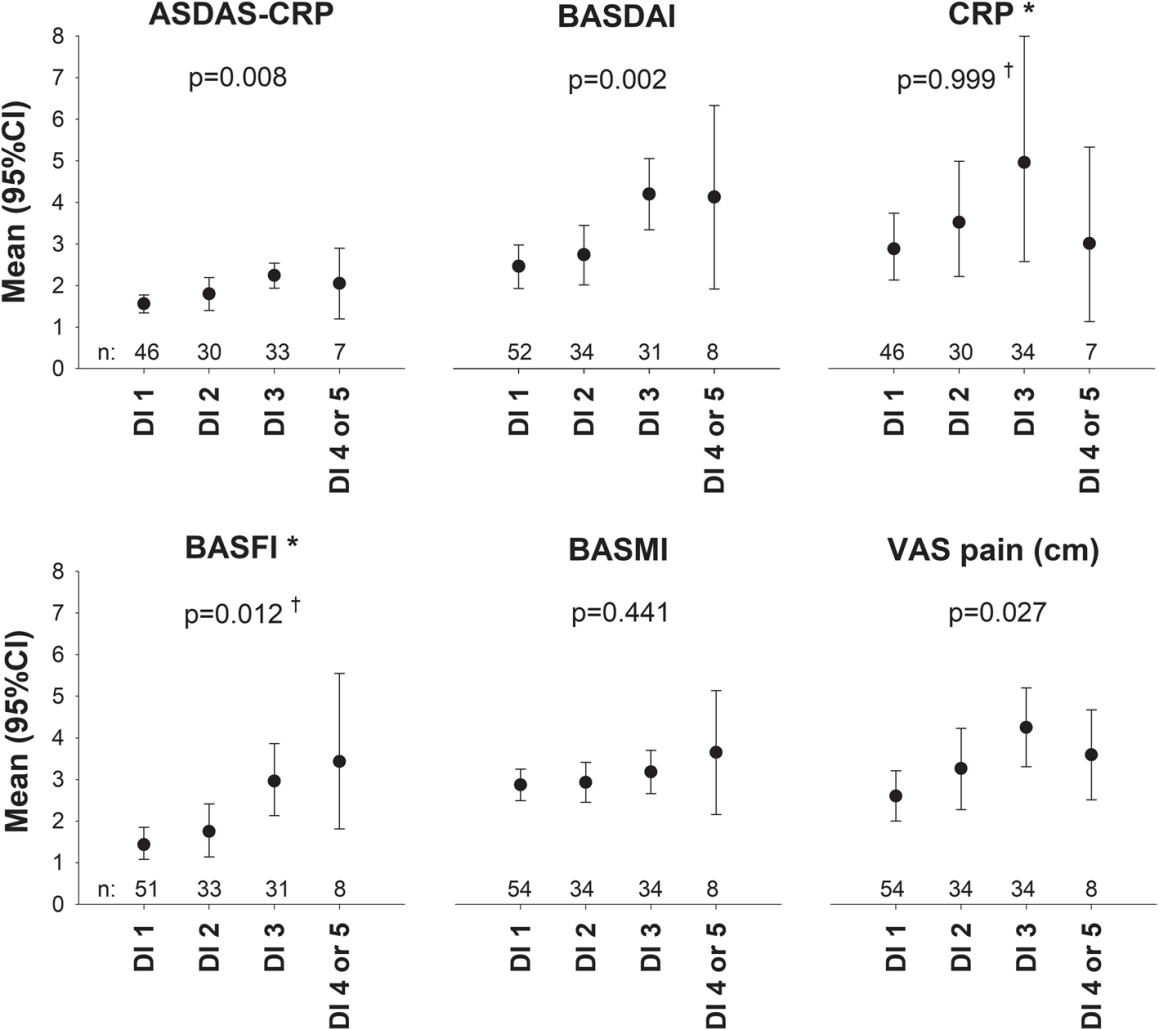
AxSpA measures in relation to dysbiosis index levels. Distributions of measures of disease activity, physical function, mobility, and pain among axSpA patients (nr-axSpA and AS combined) with different levels of dysbiosis index (DI) score 1–5. Number of analyzed subjects per group presented above the *x*-axes (DI 4 and 5 combined into one group due to too few cases). *P* values for overall between-group differences by one-way ANOVA (or Kruskal-Wallis test ^†^) displayed in the graphs. *Bootstrapped 95% CI. ANOVA analysis of variance, AS ankylosing spondylitis, axSpA axial spondyloarthritis, ASDAS-CRP ankylosing spondylitis disease-activity score using C-reactive protein, BASDAI Bath ankylosing spondylitis disease activity index, BASFI Bath ankylosing spondylitis functional index, BASMI Bath ankylosing spondylitis metrology index, CI confidence interval, CRP C-reactive protein, DI dysbiosis index, nr-axSpA non-radiographic axial spondyloarthritis, VAS visual analog scale

**Fig. 3:**
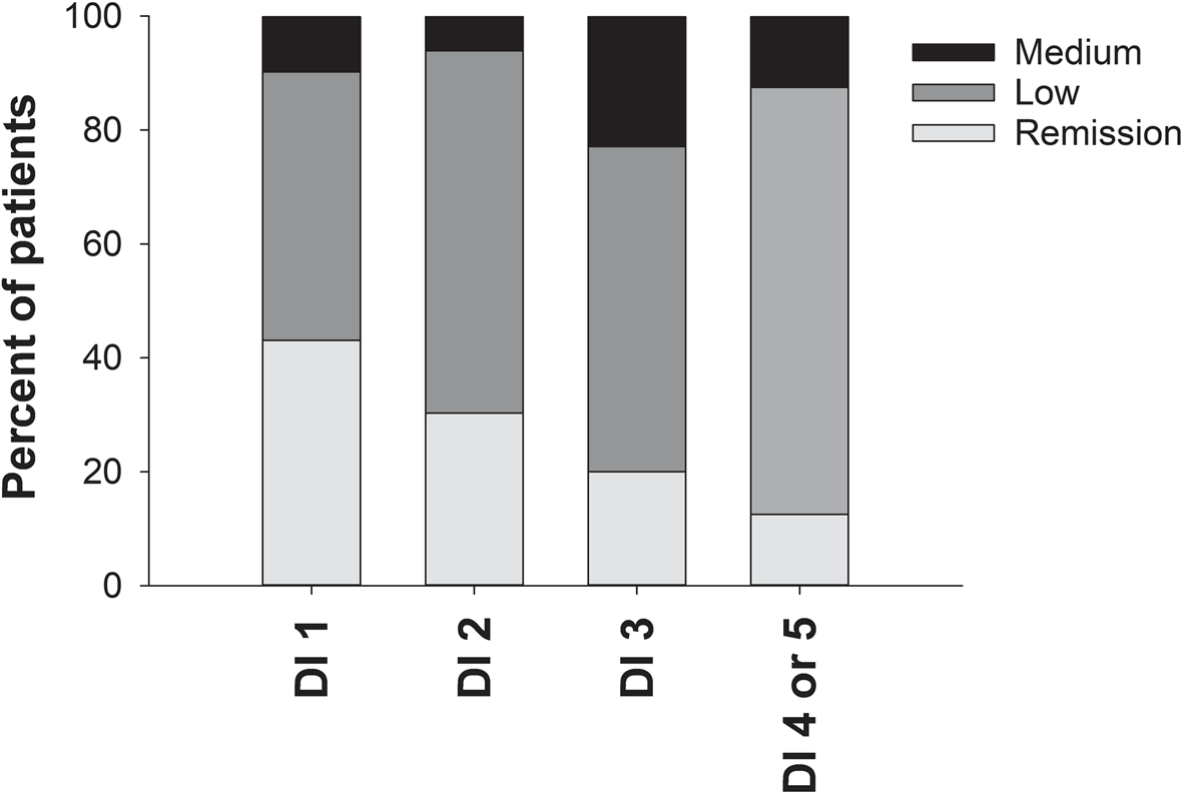
Evaluator’s global assessment of disease activity in relation to dysbiosis index levels. Distribution of Evaluator’s global assessment of disease activity (EvalGlobal; Likert scale 0–4, corresponding to Remission/Low/Medium/High/Maximal) among axSpA patients (nr-axSpA and AS combined) with different levels of dysbiosis index (DI) score 1–5. Number of patients in each group: DI 1 *n*=51, DI 2 *n*=33, DI 3 *n*=35, DI 4, or 5 *n*=8 (DI 4 and 5 combined into one group due to too few cases). No patients in any DI group were assessed as having high or maximal disease activity. *P*=0.055 for overall between-group difference by Kruskal-Wallis test. AS ankylosing spondylitis, axSpA axial spondyloarthritis, DI dysbiosis index, nr-axSpA non-radiographic axial spondyloarthritis

Among the axSpA patients (nr-axSpA and AS combined), higher DI score 1–5 (combining DI 4 and 5 into one group, due to too few cases) was associated with worse scores of most assessed axSpA measures (Fig. 2). Significant overall between-group differences across the DI score 1–5 categories were found for ASDAS-CRP, BASDAI, BASFI, and VAS pain, but not for EvalGlobal, CRP, or BASMI (Figs. 2 and **3**; *p*=0.055 for EvalGlobal).

### Differences in axSpA measures in patients with versus without gut dysbiosis

When instead splitting the axSpA group (nr-axSpA and AS combined) according to presence of gut dysbiosis (DI <3 [*n*=89] versus ≥3 [*n*=43]), patients displaying dysbiosis had higher BMI and were more often ongoing or prior smokers (Table 2). HLA-B27 positivity was less frequent in the dysbiotic group, while gut inflammation (F-calprotectin ≥50 mg/kg) was numerically and IBS symptoms significantly more common in subjects with dysbiosis (Table 2).

**Table 2:** Characteristics of the axSpA patients (nr-axSpA and AS combined), stratified by the presence of gut dysbiosis

	AxSpA without dysbiosis (DI <3) *n*=89	AxSpA with dysbiosis (DI ≥3) *n*=43
**Male sex,** ***n*** **(%)**	49 (55%)	23 (54%)
**Age, years**	51 (13)	55 (13)
**BMI, kg/m**^**2**^	26 (3.9)	28 (4.7) ‡‡
**Smoking status**		
Never smoker, *n* (%)	67 (75%)	2 (51%)
Quit smoking >6 months ago, *n* (%)	17 (19%)	14 (33%)
Intermittent smoker or quit <6 months ago, *n* (%)	1 (1.1%)	4 (9.3%)
Every day smoker, *n* (%)	4 (4.5%)	3 (7.0%)
**Symptom duration, years**	25 (13)	28 (15)
**HLA-B27 positive,** ***n*** **(%)**	83 (94%)	31 (72%) ‡
**Nr-axSpA,** ***n*** **(%)**	33 (37%)	11 (26%)
**F-calprotectin, mg/kg**		
Mean (SD)	56 (71)	112 (206)
Median (IQR)	30 (55)	39 (69)
**Elevated F-calprotectin ≥50 mg/kg,** ***n*** **(%)**	29 (33%)	17 (41%)
**IBS symptoms,** ***n*** **(%)**^a^	22 (25%)	21 (49%) ‡
**ASDAS-CRP**	1.7 (0.9)	2.2 (0.9) ‡
**BASDAI**	2.6 (2.0)	4.2 (2.4) ‡
**EvalGlobal, 0–4, median (IQR)**^b^	1 (0-1)	1 (1-1) ‡
**CRP, mg/l**	3.1 (3.3)	4.6 (7.6)
**BASFI**	1.6 (1.6)	3.1 (2.5) ‡
**BASMI**	2.9 (1.4)	3.3 (1.5)
**VAS pain, cm**	2.9 (2.5)	4.1 (2.5) ‡
**ASAS 3-month NSAID score**	35 (41)	39 (50)
**Use of proton-pump inhibitors last 3 months,** ***n*** **(%)**	33 (37%)	20 (47%)
**Ongoing csDMARD,** ***n*** **(%)**	17 (19%)	7 (16%)
Methotrexate, *n* (%)	10 (11.2%)	4 (9.3%)
Sulfasalazine, *n* (%)	5 (5.6%)	3 (7.0%)
Other csDMARD, *n* (%)	2 (2.2%)	0 (0%)
**Ongoing bDMARD,** ***n*** **(%)**	37 (42%)	19 (44%)
Adalimumab, *n* (%)	8 (9.0%)	2 (4.7%)
Certolizumab pegol, *n* (%)	7 (7.9%)	4 (9.3%)
Etanercept, *n* (%)	14 (15.7%)	5 (11.6%)
Golimumab, *n* (%)	3 (3.4%)	2 (4.7%)
Infliximab, *n* (%)	4 (4.5%)	5 (11.6%)
Secukinumab, *n* (%)	1 (1.1%)	1 (2.3%)

Mean (SD) if not otherwise stated. ^a^Meeting the ROME III criteria for IBS. ^b^Likert scale 0–4, corresponding to Remission/Low/Medium/High/Maximal. ‡*p*<0.05 for between-group difference between axSpA patients (nr-axSpA and AS combined) with versus without gut dysbiosis by chi^2-^test/Fisher’s exact test or Mann-Whitney *U* test, as appropriate. Missing data, *n* (%): Symptom duration 1 (0.8%); HLA-B27 1 (0.8%); F-calprotectin 2 (1.5%); IBS symptoms 1 (0.8%); ASDAS-CRP 16 (12%); BASDAI 7 (5.3%), EvalGlobal 5 (3.8%); CRP 15 (11%); BASFI 9 (6.8%); BASMI 2 (1.5%); VAS pain 2 (1.5%). *AS* ankylosing spondylitis, *ASDAS-CRP* ankylosing spondylitis disease activity score using CRP, *ASAS* Assessment of SpondyloArthritis international Society, *AxSpA* axial spondyloarthritis, *BASDAI* Bath ankylosing spondylitis disease activity index, *BASFI* Bath ankylosing spondylitis functional index, *BASMI* Bath ankylosing spondylitis metrology index, *bDMARD* biologic disease-modifying anti-rheumatic drug, *CRP* C-reactive protein, *csDMARD* conventional synthetic disease-modifying anti-rheumatic drug, *DI* dysbiosis index, EvalGlobal Evaluator’s global assessment of disease activity, *IBS* irritable bowel syndrome, *IQR* interquartile range, *nr-axSpA* non-radiographic axial spondyloarthritis, *NSAID* non-steroidal anti-inflammatory drug, *SD* standard deviation, *VAS* visual analog scale

**Fig. 4:**
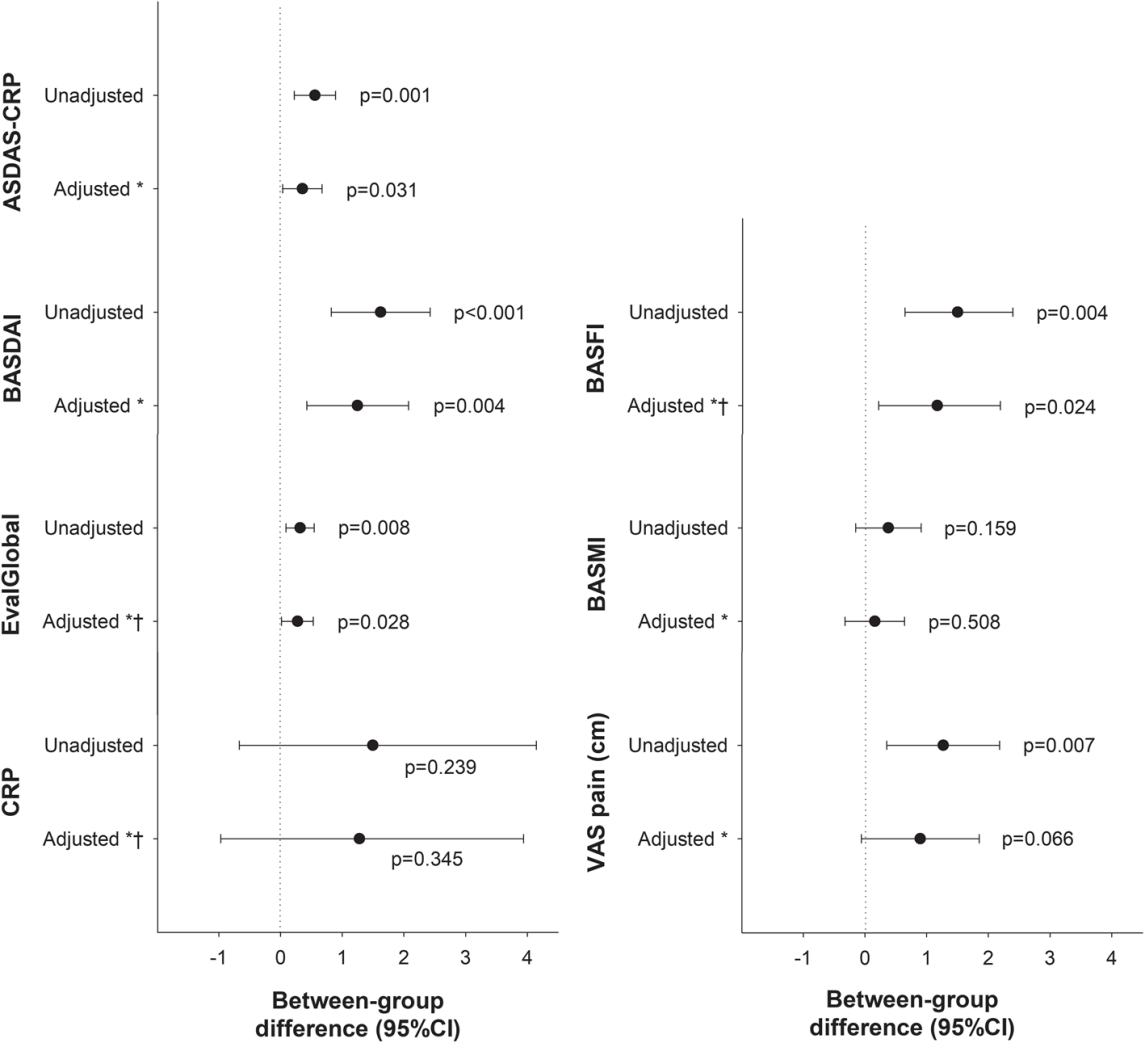
Differences in axSpA measures in patients with versus without gut dysbiosis. Differences in measures of disease activity, physical function, mobility, and pain between axSpA patients (nr-axSpA and AS combined) with gut dysbiosis (DI ≥3) versus those without dysbiosis (DI <3). The data shown represent point-estimate differences (dots) with 95% CI (whiskers) and corresponding *p* values from unadjusted analyses, and after adjustment (*) for age, sex, BMI, smoking, axSpA subtype (nr-axSpA/AS), HLA-B27 status (positive/negative), ongoing anti-TNF therapy (yes/no), ASAS 3-month NSAID score, gut inflammation (F-calprotectin ≥50 mg/kg, yes/no), and IBS-symptoms (yes/no) (ANCOVA). ^†^ Bootstrapped 95% CI. ANCOVA analysis of covariance, AS ankylosing spondylitis, ASAS Assessment of SpondyloArthritis international Society, axSpA axial spondyloarthritis, ASDAS-CRP ankylosing spondylitis disease-activity score using C-reactive protein, BASDAI Bath ankylosing spondylitis disease activity index, BASFI Bath ankylosing spondylitis functional index, BASMI Bath ankylosing spondylitis metrology index, BMI body mass index, CI confidence interval, CRP C-reactive protein, DI dysbiosis index, EvalGlobal Evaluator’s global assessment of disease activity, F fecal, HLA human leukocyte antigen, IBS irritable bowel syndrome, nr-axSpA non-radiographic axial spondyloarthritis, NSAID non-steroidal anti-inflammatory drug, TNF tumor necrosis factor, VAS visual analog scale

Comparing the axSpA measures between patients with versus without gut dysbiosis, univariate analyses showed significantly higher ASDAS-CRP, BASDAI, BASFI, VAS pain, and EvalGlobal scores for patients with versus without dysbiosis (Fig. 4; Table 2). These between-group differences remained significant also after adjustment, except for VAS pain (*p*=0.066) (Fig. 4). Regarding CRP and BASMI, no differences were detected between patients with versus without dysbiosis, neither univariately nor in the adjusted analyses (Fig. 4).

In the sensitivity analyses, the comparisons displayed in Fig. 4 were repeated after having respectively limited the study population to patients without gut inflammation (F-calprotectin <50 mg/kg; *n*=84) or to those not reporting IBS symptoms (*n*=88). In both cases, between-group differences for patients with versus without dysbiosis seen in the main analysis were confirmed or found even more distinct regarding BASDAI and VAS pain, and in relation to gut inflammation also for ASDAS-CRP and BASFI (see Additional file 1, Figures S2 and S3). However, regarding EvalGlobal, no significant difference remained between the groups after adjustment in the patient sample without gut inflammation (*p*=0.061; see Additional file 1, Figure S2). Also in the sample without IBS symptoms, no between-group differences were observed for EvalGlobal in either the univariate or adjusted analysis, nor for ASDAS-CRP or BASFI in the adjusted analysis (see Additional file 1, Figure S3).

## Discussion

### Main findings

In this population-based, cross-sectional study of well-characterized axSpA patients without IBD, we found gut dysbiosis to be significantly more frequent in AS (36%) than controls (17%). Likewise, the degree of gut microbiota aberration (as measured by DI score 1–5) was significantly higher in AS than controls. Conversely, neither dysbiosis frequency nor DI score 1–5 were significantly increased in nr-axSpA compared to controls, although numerically the nr-axSpA point-estimates fell between those of the AS patients and controls.

More importantly (and to the best of our knowledge not this clearly demonstrated before), in the overall axSpA group (nr-axSpA and AS combined), a state of gut dysbiosis was associated with significantly worse disease activity, physical function, and pain as measured by ASDAS-CRP, BASDAI, EvalGlobal, BASFI, and VAS pain. The associations remained also after adjustment for relevant confounders, including gut inflammation, anti-TNF, and NSAID treatment, except regarding VAS pain (where statistical significance was just barely lost). Restricting the analyses to include only patients without gut inflammation (as measured by elevated F-calprotectin ≥50 mg/kg) further demonstrated the associations to be present regardless of such inflammation. Thus, similar to prior observations regarding gut inflammation in axSpA [10, 25–28], but seemingly independent of such inflammation, gut dysbiosis may be a marker of more severe axSpA.

### Previous research

#### Gastrointestinal pathology, including dysbiosis, and the pathogenesis of SpA

Gut dysbiosis is believed to play an important role in IBD pathogenesis through complex and incompletely understood interactions with genetic and environmental risk factors, gut barrier defects, and abnormal immune responses [13]. SpA is closely associated with IBD—in particular Crohn’s disease—both genetically and in terms of clinical comorbidity, and many of their shared genetic risk loci govern mucosal immune defenses [7]. Furthermore, several pathological, gastrointestinal processes and aberrations reminiscent of those in IBD have been observed also in SpA, including gut dysbiosis [18–24, 40], gut inflammation (present in 50–60% of SpA patients, although subclinical in most cases) [8–10], and signs of gut barrier defects [41]. Based on this, one central hypothesis for SpA pathogenesis implicates such IBD-like, gastrointestinal processes, including dysbiosis, as an upstream event, subsequently inducing an IL-23/IL-17-pathway driven inflammation of the spine and joints [6, 7, 42, 43]. The present results, linking gut dysbiosis to worse axSpA phenotype and more active disease appear to fit well with such a theory. This hypothesis remains unproven, however, and the close links between IBD/IBD-like pathology and SpA may also be due to their shared genetic predisposition, without a causal relationship [44]. Regardless of which, ongoing gut inflammation has been repeatedly associated with more severe SpA [10, 25–28], demonstrating an important interaction between the gut and the musculoskeletal apparatus in this disease.

#### Gut dysbiosis in axSpA

The current results are congruent with previous findings which have repeatedly associated AS with altered gut microbiota [18–23], whereas little prior information is available regarding nr-axSpA [24]. While compositional analyses of the gut microbiome have consistently been able to distinguish AS from healthy controls [18–21, 23], the specific microbiota alterations observed have been heterogeneous, and no specific changes at any level of the bacterial taxonomic hierarchy have so far been identified as typical for axSpA. Furthermore, several features reminiscent of the gut dysbiosis of IBD have been reported in AS [18–21, 40]. Compositional analyses, however, clearly distinguish the gut microbiome of AS from that of IBD [19, 23], showing that despite certain similarities, the type of dysbiosis in the two diseases is not the same.

As outlined above, 50–60% of SpA patients display microscopic gut inflammation [8–10], but whether there is a causal relationship between this and gut dysbiosis—and if so, in which direction—remains incompletely understood. The two processes are, however, intertwined, since the presence of inflammation has been associated with a significantly different gut microbiota composition compared to SpA patients without histological gut inflammation [24]. Furthermore, another study showed the gut microbiota composition to differ between AS patients with elevated versus normal F-calprotectin [19].

In the present study, gut microbiota aberrations were assessed by the validated, semi-quantitative DI of the GA-map™ Dysbiosis Test. Klingberg et al. previously applied the same method in a cohort of 150 AS patients, finding a substantially higher gut dysbiosis (DI ≥3) prevalence of 87% [19], as compared to the 36% in our AS sample. Of interest in light of our results, however, their cohort also displayed higher disease activity and worse physical function, with median BASDAI/BASFI scores of 3.2/2.3 versus 2.2/1.2 in our AS group, and thus represented a patient group with worse disease.

#### AxSpA disease severity in relation to gut dysbiosis

To our knowledge, only a few studies have previously investigated gut microbiota aberrations in SpA in relation to disease severity, most seeming to indicate an association with disease activity in line with our results. Tito et al. studied the intestinal microbiota in biopsies from 27 axSpA patients (AS and nr-axSpA) and found a significant positive correlation between the abundance of the genus *Dialister* and disease activity as measured by ASDAS (*r*_s_=0.62), and a similar trend for BASDAI (*r*_s_=0.53) [24]. In another study, Breban et al. observed an enrichment in *Ruminococcus gnavus* in SpA, which was significant in relation to controls only for cases with active disease (BASDAI ≥3) [40]. This enrichment in association with higher SpA disease activity was observed regardless of concomitant IBD, but the elevation was more pronounced in those with comorbid IBD, for whom there was also a significant positive correlation between *Ruminucoccus gnavus* abundance and BASDAI (*r*_s_=0.77). Finally, a recent abstract from a multinational study on gut microbiota in AS showed that the bacterial composition differed significantly between patients with different BASDAI levels (categorized as <2.5/2.5-5/5-7.5/>7.5), congruent with our findings [23].

Conversely, Klingberg et al. reported no associations between gut microbiota composition, assessed by the GA-map™ Dysbiosis Test, and ASDAS-CRP, BASDAI, BASFI, BASMI, CRP, or ESR (erythrocyte sedimentation rate) [19]. However, in contrast to our analyses, their study did not relate these measures to the DI, but performed compositional analysis, comparing the gut microbiota composition between patient groups with better or worse (dichotomized) levels of the different indices. Notably, as stated above, Klingberg et al. found dysbiosis (DI ≥3) in as many as 87% of their AS patients (*n*=130 versus *n*=20 without dysbiosis), a distribution which may have limited their ability to detect differences in disease measures based on microbiota composition.

Apart from excluding [19, 24] or separately analyzing [40] patients with comorbid IBD, none of the above-cited studies [19, 23, 24, 40] adjusted their analyses regarding the link between gut microbiota aberrations and disease severity for intestinal inflammation. Thus, some reported observations may have been driven by the known link between gut inflammation and more severe disease [10, 25–28], rather than by the dysbiosis per se. Here, we both adjusted for F-calprotectin and performed a sensitivity analysis excluding all patients with elevated F-calprotectin (≥50 mg/kg), aiming to more specifically assess a potential association with gut dysbiosis. In this context, it should be acknowledged that the ability of a F-calprotectin <50 mg/kg to rule out asymptomatic, microscopic (histological) gut inflammation, which is much more common in SpA than overt IBD [8, 27], has not been widely studied, and F-calprotectin may also be less sensitive for small-bowel than colonic inflammation [45]. Cypers et al., however, did report significantly elevated F-calprotectin in SpA patients displaying microscopic gut inflammation in the terminal ileum and/or colon, with an optimal F-calprotectin cut-off of 85 mg/kg for the detection of such cases [46]. Furthermore, the presence of macroscopic inflammation at capsule endoscopy and/or ileocolonoscopy in SpA patients with previously undiagnosed IBD has also been shown to be significantly associated with elevated F-calprotectin levels (>100 mg/kg) [47, 48]. Taken together, the current results seem to indicate that gut dysbiosis may be associated with worse axSpA disease activity and physical function, independently of intestinal inflammation.

Similarly, since IBS symptoms have previously been associated with worse patient-reported axSpA measures in the SPARTAKUS cohort [38] and that gut dysbiosis is intrinsic to IBS [39], the same adjustment and sensitivity analysis approach was also applied for IBS symptoms, again without relevant changes to our results. Anti-TNF therapy, used by 41% of our patients, has previously shown a potential to ameliorate gut dysbiosis in axSpA [20, 36] and was therefore adjusted for, without any considerable impact on the findings. Moreover, proton-pump inhibitor (PPI) use was considered as an additional adjustment factor due to its described association with gut dysbiosis [49], but was omitted since PPI-use during the last 3 months did not differ significantly between patients with versus without dysbiosis (DI ≥3) in our material (*p*=0.300 by chi^2^-test) and since no causal relationship between PPI use and the axSpA measures was suspected.

### Strengths and limitations

This study provides new knowledge regarding how gut dysbiosis relates to axSpA phenotype and disease measures (regarding disease activity, function, mobility, and pain)—associations previously investigated by only a limited number of studies. Patients were consecutively enrolled from a population-based cohort study and were well characterized and classified according to the ASAS axSpA and modified New York criteria. The detailed protocol allowed for extensive adjustment for possible confounders, including BMI, smoking, axSpA subtype, HLA-B27 status, anti-TNF and NSAID therapy, intestinal inflammation, and IBS symptoms. By including F-calprotectin, we could—to a certain degree—disentangle a link between gut dysbiosis and worse axSpA disease activity and function from the impact of simultaneous intestinal inflammation.

Another strength is that, in addition to AS, our study also encompasses nr-axSpA, a disease subtype for which data regarding gut microbiota are scant. The relatively low number of nr-axSpA patients (*n*=44) means that we cannot rule out type 2 error from contributing to the lack of significant differences between this group and both AS and controls. Yet, the pattern by which the nr-axSpA point-estimates fall in between those of AS and controls is similar to prior observations for F-calprotectin [27]. The relatively long mean symptom duration of our nr-axSpA group should also be remembered when assessing the results, since it likely entails a lower average risk of future progression to AS than in a cohort of newly diagnosed nr-axSpA.

Regarding generalizability, only comorbid IBD and recent antibiotic use were exclusion criteria. Without restrictions regarding disease subtype (nr-axSpA or AS) or severity, treatment, or other comorbidities, we believe our results to be fairly generalizable to the wider axSpA population. However, in the absence of any concurrent ICD-10 codes for SpA (usually added in case of axial involvement), patients clinically diagnosed with psoriatic, IBD-associated, or reactive arthritis (by ICD-10 codes) were not included, although these conditions may sometimes have axial disease.

Regarding limitations, endoscopic examinations and biopsies were not part of the SPARTAKUS protocol, but could have provided valuable information regarding histological inflammation and mucosa-associated microbiota (fecal and mucosa-associated microbiota have been shown to differ [50, 51]). The use of deep-sequencing or shotgun metagenomics sequencing methods, rather than the pre-determined target approach of the GA-map™ Dysbiosis Test, would have provided a much more detailed characterization of the gut microbiota. Assessment of associations between specific bacteria and axSpA disease measures were, however, beyond the scope of the present study. For the determination of gut dysbiosis, the validated GA-map™ Dysbiosis Test has shown good agreement with deep-sequencing methodology [31], and by the DI, it provides a readily comprehensible, semiquantitative score of global dysbiosis, calibrated to healthy individuals as reference of normobiosis. Finally, information regarding probiotics use is missing, and the cross-sectional design did not allow for longitudinal evaluation of microbiota shifts or disease development.

## Conclusions

The presence of gut dysbiosis is linked to worse axSpA phenotype and more active disease. While dysbiosis was more prevalent in AS than controls, no significant difference was observed between nr-axSpA and controls. In the overall axSpA group (nr-axSpA and AS combined), the presence of gut dysbiosis was independently associated with worse disease activity and physical function, seemingly irrespective of both gut inflammation and ongoing treatments. Further research is needed to assess the validity of these findings also in other axSpA cohorts, as well as to gain a deeper understanding of the connection between gut microbiota and axSpA, and examine whether a causal relationship exists. This would offer guidance as to whether targeting the intestine therapeutically, with regard to the luminal microbiota composition and/or mucosal inflammation, could be a relevant approach to try to interfere with axSpA development or mitigate the disease course. Nonetheless, the current results add to the steadily growing body of evidence for an important, potentially pathobiological, link between disturbances in gastrointestinal homeostasis and axSpA.

## Supplementary Information

**Additional file 1. **Exploratory analyses of bacterial abundance in axial spondyloarthritis patients versus controls, and sensitivity analyses regarding disease activity, physical function, mobility and pain in relation to gut dysbiosis status in the patient group. Exploratory analyses comparing probe signal intensity for the 48 bacterial markers of the GA-map™ Dysbiosis Test between the axial spondyloarthritis patients and controls (including **Figure S1**). **Figure S2.** Differences in measures of disease activity, physical function, mobility and pain between axial spondyloarthritis patients with gut dysbiosis versus those without dysbiosis, when only including patients without gut inflammation. **Figure S3.** Differences in measures of disease activity, physical function, mobility and pain between axial spondyloarthritis patients with gut dysbiosis versus those without dysbiosis, when only including patients without irritable bowel syndrome symptoms.

## Data Availability

The datasets used and analyzed for the current study are available from the corresponding author on reasonable request.

## Abbreviations

ANCOVA: Analysis of covariance
AS: Ankylosing spondylitis
ASAS: Assessment of SpondyloArthritis international Society
ASDAS-CRP: Ankylosing spondylitis disease activity score using CRP
axSpA: Axial spondyloarthritis
BASDAI: Bath ankylosing spondylitis disease activity index
BASFI: Bath ankylosing spondylitis functional index
BASMI: Bath ankylosing spondylitis metrology index
bDMARD: Biologic disease-modifying anti-rheumatic drug
BMI: Body mass index
CI: Confidence interval
CRP: C-reactive protein
csDMARD: Conventional synthetic disease-modifying anti-rheumatic drug
DI: Dysbiosis index
DMARD: Disease-modifying anti-rheumatic drug
ESR: Erythrocyte sedimentation rate
EvalGlobal: Evaluator’s global assessment of disease activity
HLA-B27: Human leukocyte antigen B27
IBD: Inflammatory bowel disease
IBS: Irritable bowel syndrome
IL: Interleukin
IQR: Interquartile range
mNY: Modified New York criteria
Nr-axSpA: Non-radiographic axial spondyloarthritis
NSAID: Non-steroidal anti-inflammatory drug
PPI: Proton-pump inhibitor
SD: Standard deviation
TNF: Tumor necrosis factor
VAS pain: Visual analog scale for pain
