# Supplementary material for: Gut dysbiosis associated with worse disease activity and physical function in axial spondyloarthritis
Source: Arthritis Res Ther. 2022 Feb 12;24:42. doi: 10.1186/s13075-022-02733-w (PMC8840679; doi:10.1186/s13075-022-02733-w)
Supplement: Supplementary file 1 — Additional file 1. Exploratory analyses of bacterial abundance in axial spondyloarthritis patients versus controls, and sensitivity analyses regarding disease activity, physical function, mobility and pain in relation to gut dysbiosis status in the patient group. Exploratory analyses comparing probe signal intensity for the 48 bacterial markers of the GA-map™ Dysbiosis Test between the axial spondyloarthritis patients and controls (including Figure S1). Figure S2. Differences in measures of disease activity, physical function, mobility and pain between axial spondyloarthritis patients with gut dysbiosis versus those without dysbiosis, when only including patients without gut inflammation. Figure S3. Differences in measures of disease activity, physical function, mobility and pain between axial spondyloarthritis patients with gut dysbiosis versus those without dysbiosis, when only including patients without irritable bowel syndrome symptoms. [file 13075_2022_2733_MOESM1_ESM.docx]

**Additional file 1**

**_________________________________________________________**

**Gut dysbiosis associated with worse disease activity and**

**physical function in axial spondyloarthritis**

Jonas Sagard^1,2^, Tor Olofsson^1,2^, Elisabeth Mogard^1,2^, Jan Marsal^3,4^, Kristofer Andréasson^1,2^, Mats Geijer^5,6,7^, Lars Erik Kristensen^1,8^, Elisabet Lindqvist^1,2^, Johan K Wallman^1,2^

^1^ Section of Rheumatology, Department of Clinical Sciences Lund, Lund University, Lund, Sweden

^2^ Skåne University Hospital, Department of Rheumatology, Lund, Sweden

^3^ Department of Immunology, EMV, Lund University, Lund, Sweden

^4^ Department of Gastroenterology, Skåne University Hospital, Lund/Malmö, Sweden

^5^ Sahlgrenska Academy, Institute of Clinical Sciences, Department of Radiology, University of Gothenburg, Gothenburg, Sweden

^6^ Region Västra Götaland, Department of Radiology, Sahlgrenska University Hospital, Gothenburg, Sweden

^7^ Section of Radiology, Department of Clinical Sciences Lund, Lund University, Lund, Sweden

^8^ Parker Institute, Frederiksberg and Bispebjerg, Department of Rheumatology, Copenhagen University Hospital, Copenhagen, Denmark

**Table of contents:**

Page 3: Exploratory analyses comparing probe signal intensity for the 48 bacterial markers of the GA-map™ Dysbiosis Test between the axial spondyloarthritis patients and controls (including Figure S1).

Page 6: Figure S2. Differences in measures of disease activity, physical function, mobility and pain between axial spondyloarthritis patients with gut dysbiosis versus those without dysbiosis, when only including patients without gut inflammation.

Page 8: Figure S3. Differences in measures of disease activity, physical function, mobility and pain between axial spondyloarthritis patients with gut dysbiosis versus those without dysbiosis, when only including patients without irritable bowel syndrome symptoms.

Page 10: References

**Exploratory analyses comparing probe signal intensity for the 48 bacterial markers of the GA-map™ Dysbiosis Test between the axial spondyloarthritis patients and controls**

To assess the nature of gut dysbiosis in our patients, exploratory analyses comparing the probe signal intensities for the 48 bacterial markers (at different taxonomic levels) included in the GA-map™ Dysbiosis Test between the axial spondyloarthritis (axSpA) patients (non-radiographic axSpA [nr-axSpA] and ankylosing spondylitis [AS] combined) and controls were performed.

*Results*

As displayed in **Figure S1**, at the genus level (phylum within brackets) *Escherichia/Shigella* (Proteobacteria), *Streptococcus* (Firmicutes) and *Lactobacillus* (Firmicutes) species were found to be more abundant in axSpA than in controls. A similar trend was also seen for *Bacteroides* (Bacteroidetes) species. At class level, Bacilli (Firmicutes) was more abundant in axSpA, while at the phylum level, a trend towards elevation in axSpA was also observed regarding Proteobacteria (p=0.066, despite the 95% CI not overlapping zero).

*Discussion*

Although previous studies have linked AS with gut dysbiosis, no uniform dysbiotic features considered characteristic of axSpA have as yet become clear. Nonetheless, altered abundances regarding the above mentioned bacteria have also been reported from some prior studies.

Of note, our results show clear similarities with the gut dysbiosis observed in another Swedish cohort of axSpA (only including AS patients, and applying the same GA-map™ Dysbiosis Test as in the present study), where increased abundances in relation to controls were seen for Proteobacteria, *Enterobacteriaceae* and *Escherichia/Shigella* species*,* as well as for Bacilli, including specifically for *Lactobacillus* and *Streptococcus* species (1). However, in contrast to our results, they also showed higher abundance of Actinobacteria, and lower abundance of certain *Bacteroides* species and *Lachnospiraceae* (1).

Looking further, other studies have also found an increased abundance of the phylum Proteobacteria and of *Escherichia/Shigella* species, belonging to this phylum, in AS compared with healthy controls (2, 3). Interestingly, an increased abundance of Proteobacteria, its family *Enterobacteriaceae* and species of *Escherichia* have also been found in IBD (4-7). Proteobacteria and *Escherichia coli* hold a pro-inflammatory potential, and have been demonstrated to induce experimental colitis (8, 9). There is also evidence that *Lactobacilli* may be increased in IBD, as well as in rheumatoid arthritis (5, 10). Other studies have also found a higher abundance of *Streptococcus* (2, 11, 12) and *Bacteroides* (11) species in AS compared to healthy controls.

Yet, it remains unclear why the abundances of these particular bacteria are altered and what role they may play in axSpA. Experimental research on germfree, HLA-B27 transgenic rats has showed that reintroduction of commensal microbiota was able to trigger arthritis and colitis, and that *Bacteroides* species were especially pro-inflammatory (13). Experimental recolonization with bacteria in germfree rats with adjuvant-induced arthritis has also found that *Lactobacilli* may induce a more severe disease, while *Escherichia coli* in this setting appeared to be protective (14).


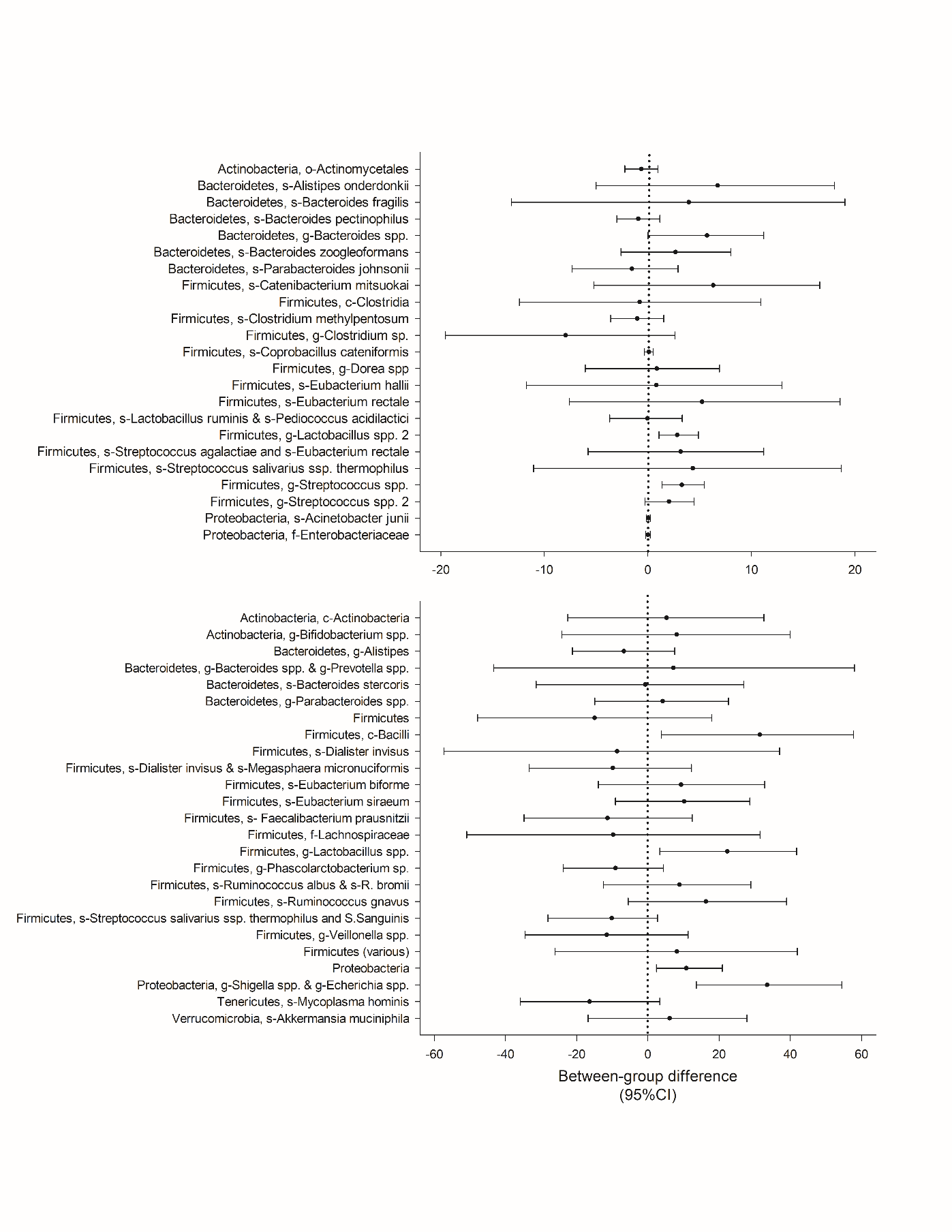


**Figure S1. Differences in probe signal intensity for the 48 bacterial markers of the GA-map™ Dysbiosis Test between axSpA patients (nr-axSpA and AS combined; n=132) and controls (n=46).** The data shown represent point-estimate differences (dots) with bootstrapped 95% CI (whiskers) from Welch´s t-tests. The bacterial markers, at different taxonomic levels, are described by Phylum, followed by c-class, o-order, f-family, g-genus or s-species. Note that probe signal intensity values are not comparable between the different bacterial markers. Since these analyses were considered exploratory, no correction for multiple testing was performed. CI, confidence interval; sp./spp., species (singular/plural).


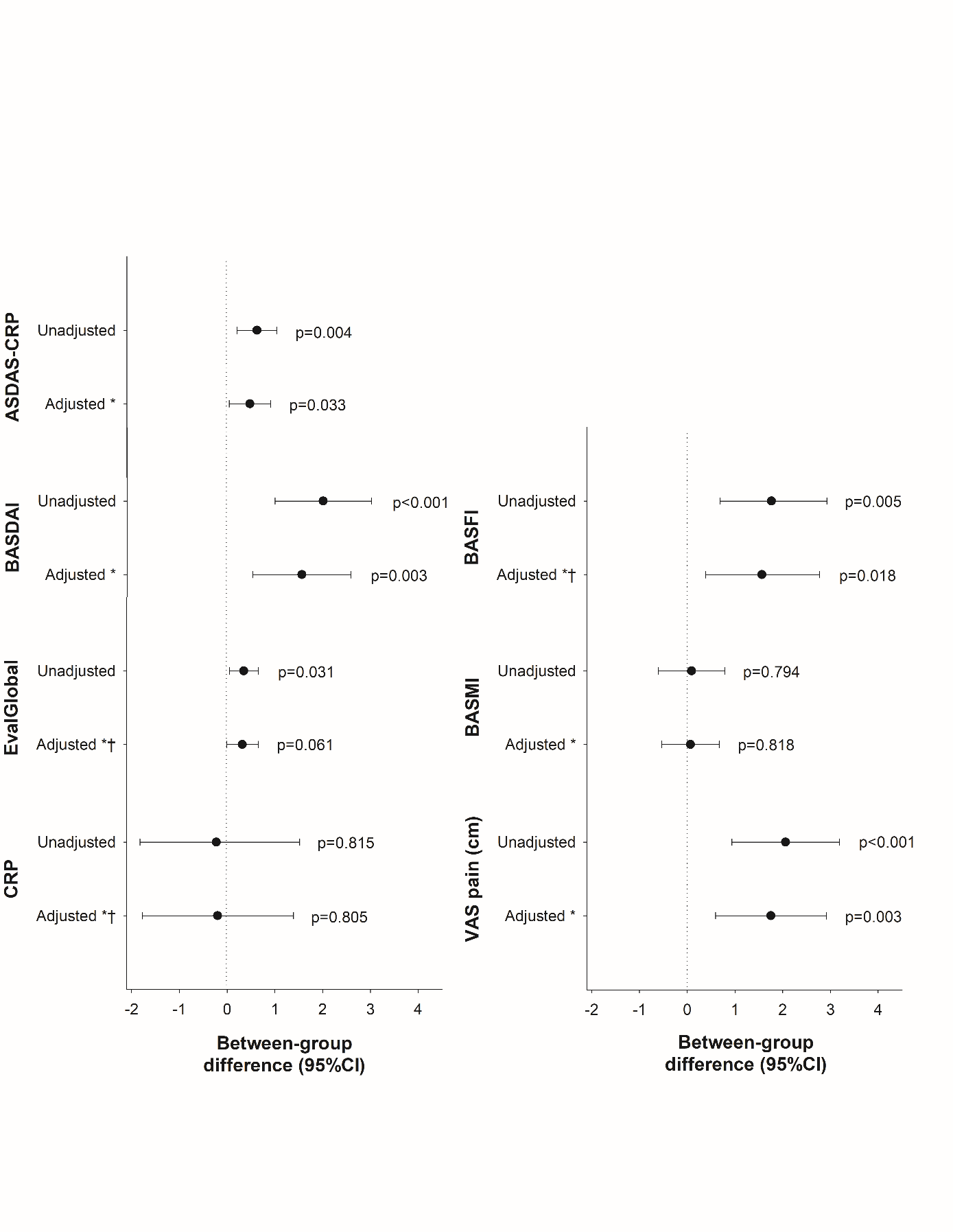


**Figure S2.** **Differences in measures of disease activity, physical function, mobility, and pain between axSpA patients (nr-axSpA and AS combined) with gut dysbiosis (DI ≥3) versus those without dysbiosis (DI <3), when only including patients without gut inflammation (i.e. with F-calprotectin <50 mg/kg; n=84).** The data shown represent point-estimate differences (dots) with 95% CI (whiskers) and corresponding p-values from unadjusted analyses, and after adjustment (*) for age, sex, BMI, smoking, axSpA subtype (nr-axSpA/AS), HLA-B27 status (positive/negative), ongoing anti-TNF therapy (yes/no), ASAS 3-month NSAID score and IBS-symptoms (yes/no) (ANCOVA). ^†^ Bootstrapped 95% CI. ANCOVA, analysis of covariance; AS, ankylosing spondylitis; ASAS, Assessment of SpondyloArthritis international Society; axSpA, axial spondyloarthritis; ASDAS-CRP, ankylosing spondylitis disease-activity score using C-reactive protein; BASDAI, Bath ankylosing spondylitis disease activity index; BASFI, Bath ankylosing spondylitis functional index; BASMI, Bath ankylosing spondylitis metrology index; BMI, body mass index; CI, confidence interval; CRP, C-reactive protein; DI, dysbiosis index; EvalGlobal, Evaluator´s global assessment of disease activity; F, fecal; HLA, human leukocyte antigen; IBS, irritable bowel syndrome; nr-axSpA, non-radiographic axial spondyloarthritis; NSAID, non-steroidal anti-inflammatory drug; TNF, tumor necrosis factor; VAS, visual analog scale.


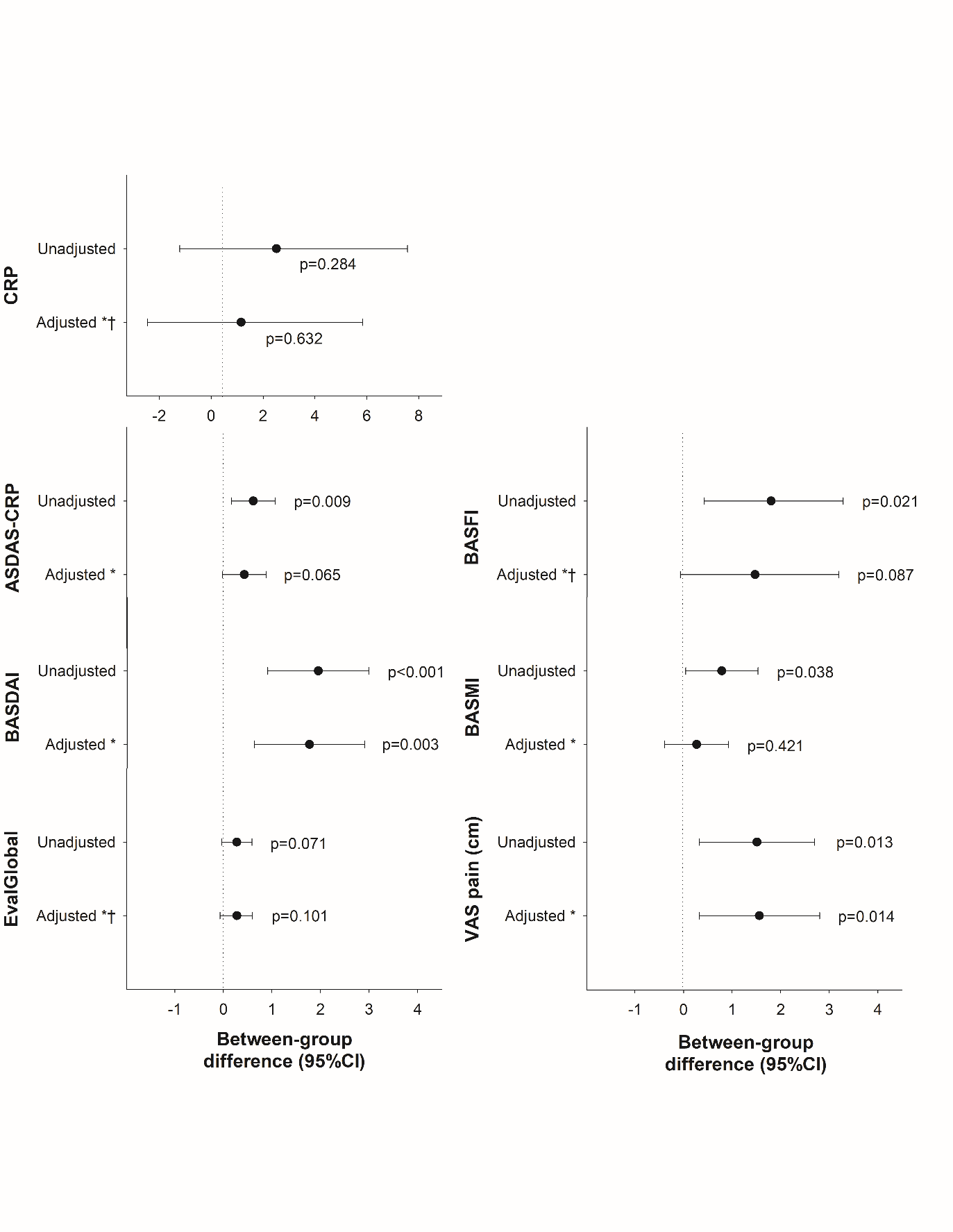


**Figure S3.** **Differences in measures of disease activity, physical function, mobility, and pain between axSpA patients (nr-axSpA and AS combined) with gut dysbiosis (DI ≥3) versus those without dysbiosis (DI <3), when only including patients without IBS symptoms (n=88).** The data shown represent point-estimate differences (dots) with 95% CI (whiskers) and corresponding p-values from unadjusted analyses, and after adjustment (*) for age, sex, BMI, smoking, axSpA subtype (nr-axSpA/AS), HLA-B27 status (positive/negative), ongoing anti-TNF therapy (yes/no), ASAS 3-month NSAID score and gut inflammation (F-calprotectin ≥50 mg/kg, yes/no) (ANCOVA). ^†^ Bootstrapped 95% CI. ANCOVA, analysis of covariance; AS, ankylosing spondylitis; ASAS, Assessment of SpondyloArthritis international Society; axSpA, axial spondyloarthritis; ASDAS-CRP, ankylosing spondylitis disease-activity score using C-reactive protein; BASDAI, Bath ankylosing spondylitis disease activity index; BASFI, Bath ankylosing spondylitis functional index; BASMI, Bath ankylosing spondylitis metrology index; BMI, body mass index; CI, confidence interval; CRP, C-reactive protein; DI, dysbiosis index; EvalGlobal, Evaluator´s global assessment of disease activity; F, fecal; HLA, human leukocyte antigen; IBS, irritable bowel syndrome; nr-axSpA, non-radiographic axial spondyloarthritis; NSAID, non-steroidal anti-inflammatory drug; TNF, tumor necrosis factor; VAS, visual analog scale.

**References**

1. Klingberg E, Magnusson MK, Strid H, Deminger A, Stahl A, Sundin J, et al. A distinct gut microbiota composition in patients with ankylosing spondylitis is associated with increased levels of fecal calprotectin. Arthritis Res Ther. 2019;21:248.

2. Li M, Dai B, Tang Y, Lei L, Li N, Liu C, et al. Altered Bacterial-Fungal Interkingdom Networks in the Guts of Ankylosing Spondylitis Patients. mSystems. 2019;4.

3. Cardoneanu A, Cozma S, Rezus C, Petrariu F, Burlui AM, Rezus E. Characteristics of the intestinal microbiome in ankylosing spondylitis. Exp Ther Med. 2021;22:676.

4. Gevers D, Kugathasan S, Denson LA, Vazquez-Baeza Y, Van Treuren W, Ren B, et al. The treatment-naive microbiome in new-onset Crohn's disease. Cell Host Microbe. 2014;15:382-92.

5. Nishino K, Nishida A, Inoue R, Kawada Y, Ohno M, Sakai S, et al. Analysis of endoscopic brush samples identified mucosa-associated dysbiosis in inflammatory bowel disease. J Gastroenterol. 2018;53:95-106.

6. Prosberg M, Bendtsen F, Vind I, Petersen AM, Gluud LL. The association between the gut microbiota and the inflammatory bowel disease activity: a systematic review and meta-analysis. Scand J Gastroenterol. 2016;51:1407-15.

7. Pittayanon R, Lau JT, Leontiadis GI, Tse F, Yuan Y, Surette M, et al. Differences in Gut Microbiota in Patients With vs Without Inflammatory Bowel Diseases: A Systematic Review. Gastroenterology. 2020;158:930-46 e1.

8. Sartor RB, Wu GD. Roles for Intestinal Bacteria, Viruses, and Fungi in Pathogenesis of Inflammatory Bowel Diseases and Therapeutic Approaches. Gastroenterology. 2017;152:327-39 e4.

9. Kittana H, Gomes-Neto JC, Heck K, Geis AL, Segura Munoz RR, Cody LA, et al. Commensal Escherichia coli Strains Can Promote Intestinal Inflammation via Differential Interleukin-6 Production. Front Immunol. 2018;9:2318.

10. Heeney DD, Gareau MG, Marco ML. Intestinal Lactobacillus in health and disease, a driver or just along for the ride? Curr Opin Biotechnol. 2018;49:140-7.

11. Zhang L, Han R, Zhang X, Fang G, Chen J, Li J, et al. Fecal microbiota in patients with ankylosing spondylitis: Correlation with dietary factors and disease activity. Clin Chim Acta. 2019;497:189-96.

12. Chen Z, Qi J, Wei Q, Zheng X, Wu X, Li X, et al. Variations in gut microbial profiles in ankylosing spondylitis: disease phenotype-related dysbiosis. Ann Transl Med. 2019;7:571.

13. Rath HC, Herfarth HH, Ikeda JS, Grenther WB, Hamm TE, Jr., Balish E, et al. Normal luminal bacteria, especially Bacteroides species, mediate chronic colitis, gastritis, and arthritis in HLA-B27/human beta2 microglobulin transgenic rats. J Clin Invest. 1996;98:945-53.

14. Kohashi O, Kohashi Y, Takahashi T, Ozawa A, Shigematsu N. Reverse effect of gram-positive bacteria vs. gram-negative bacteria on adjuvant-induced arthritis in germfree rats. Microbiol Immunol. 1985;29:487-97.
